# Time course of outcome in poor grade subarachnoid hemorrhage patients: a longitudinal retrospective study

**DOI:** 10.1186/s12883-021-02229-1

**Published:** 2021-05-13

**Authors:** Elisa Gouvêa Bogossian, Daniela Diaferia, Andrea Minini, Narcisse Ndieugnou Djangang, Marco Menozzi, Lorenzo Peluso, Filippo Annoni, Jacques Creteur, Sophie Schuind, Olivier Dewitte, Fabio Silvio Taccone

**Affiliations:** 1 Department of Intensive Care Erasmus Hospital, Université Libre de Bruxelles (ULB), Route de Lennik, 808, 1070 Brussels, Belgium; 2 Department of Neurosurgery Erasmus Hospital, Université Libre de Bruxelles (ULB), Brussels, Belgium

**Keywords:** Subarachnoid hemorrhage, Mortality, Hydrocephalus, Intracranial hemorrhage, Neurological outcome

## Abstract

**Background:**

Neurological outcome and mortality of patients suffering from poor grade subarachnoid hemorrhage (SAH) may have changed over time. Several factors, including patients’ characteristics, the presence of hydrocephalus and intraparenchymal hematoma, might also contribute to this effect. The aim of this study was to assess the temporal changes in mortality and neurologic outcome in SAH patients and identify their predictors.

**Methods:**

We performed a single center retrospective cohort study from 2004 to 2018. All non-traumatic SAH patients with poor grade on admission (WFNS score of 4 or 5) who remained at least 24 h in the hospital were included. Time course was analyzed into four groups according to the years of admission (2004–2007; 2008–2011; 2012–2015 and 2016–2018).

**Results:**

A total of 353 patients were included in this study: 202 patients died (57 %) and 260 (74 %) had unfavorable neurological outcome (UO) at 3 months. Mortality tended to decrease in in 2008–2011 and 2016–2018 periods (HR 0.55 [0.34–0.89] and HR 0.33 [0.20–0.53], respectively, when compared to 2004–2007). The proportion of patients with UO remained high and did not vary significantly over time. Patients with WFNS 5 had higher mortality (68 % vs. 34 %, p = 0.001) and more frequent UO (83 % vs. 54 %, p = 0.001) than those with WFNS 4. In the multivariable analysis, WFNS 5 was independently associated with mortality (HR 2.12 [1.43–3.14]) and UO (OR 3.23 [1.67–6.25]). The presence of hydrocephalus was associated with a lower risk of mortality (HR 0.60 [0.43–0.84]).

**Conclusions:**

Both hospital mortality and UO remained high in poor grade SAH patients. Patients with WFNS 5 on admission had worse prognosis than others; this should be taken into consideration for future clinical studies.

**Supplementary Information:**

The online version contains supplementary material available at 10.1186/s12883-021-02229-1.

## Background

Non traumatic subarachnoid hemorrhage (SAH) is a significant cause of morbidity and mortality worldwide [1]. Ruptured aneurysms are by far the most frequent cause of non-traumatic SAH [2]; despite improvement in aneurysm management and in the control of secondary brain injuries, the occurrence of long-term neurological sequalae among survivors remains high [1].

Neurological status on admission, in particular if assessed by the World Federation of Neurological Surgeons (WFNS) scale [3], is associated with poor outcome after SAH [4]. In particular, “poor grade” SAH (i.e. WFNS 4 or 5) has been consistently identified as an independent predictor of poor neurological outcome in different observational studies [5–7]. In the 70 and 80 s, only 13 % of poor grade SAH patients presented with long-term good neurological recovery [8–10]; in the following decades, good neurological recovery increased up of 30 % of admitted patients [5, 11–15]. This finding could be ascribed to several factors, such as the development of accurate diagnostic tools to rapidly identify the culprit aneurysm, the widespread use of nimodipine as prophylactic neuroprotective therapy, early treatment of the ruptured aneurysm and the development of specialized neuro-intensive care units [16]. Nevertheless, in the last 15 years, the proportion of SAH patients with poor grade on admission and who subsequently will be discharged with intact neurological outcome appears unchanged [7, 17, 18].

Secondary brain injury, especially the occurrence of delayed cerebral ischemia (DCI), is an important preventable cause of poor outcome in SAH patients [19]. The application of multimodal monitoring for early detection and the development of new and more aggressive therapeutic strategies, such as chemical or mechanical angioplasty to treat vasospasm, could contribute to the reduction of brain damage in this setting [20]. Moreover, initial poor clinical neurological status due to acute hydrocephalus may not have the same impact on neurological recovery as brain edema or large intracranial bleeding following SAH [21]. Additionally, a recent study demonstrated that patients with intraparenchymal hematoma have worse neurological outcome and should be taken into account when prognosticating poor grade SAH patients [22].

Considering the complexity of several prognostic factors in SAH patients, the primary aim of this study was to assess the temporal changes in mortality and of long-term neurologic outcome in non-traumatic SAH patients admitted to a referral center over the last 15 years. Secondary outcomes included: (a) predictors of mortality and unfavorable neurological outcome; (b) differences in the combination of WFNS 4 or 5, hydrocephalus and intraparenchymal hematoma on patients’ outcome.

## Methods

### Study population

This is a retrospective, single-center cohort study conducted in the Intensive Care Unit (ICU) of Erasmus Hospital, Brussels, Belgium. This study was approved by the local ethics committee (P2019/649), which waived the need of informed consent. All adult patients (> 18 years) admitted to the ICU after a non-traumatic SAH (i.e. including not only aneurysmal SAH but also bleeding secondary to arteriovenous malformation or *sine materia*) from January 2004 to December 2018 were eligible for screening. We included only patients with poor clinical grade at presentation, defined by a WFNS scale of 4 or 5 points [3], who remained at least 24 h in the ICU.

### Data collection

Demographic data were recorded, including sex and gender of patients, history of hypertension, chronic obstructive pulmonary disease (COPD), heart disease, liver cirrhosis, chronic renal failure, cancer, immunosuppressive therapy, and previous neurological disease. Clinical status on admission was evaluated using the Glasgow Coma Scale (GCS) [23] and the WFNS score. Severity of the disease was assessed using the acute physiology and chronic health evaluation (APACHE) II score [24] and the sequential organ failure assessment (SOFA) score on admission [25]; the severity of the initial bleeding was assessed from the initial head computed tomography (CT) scan, using the Fisher scale [26]. The type of treatment (i.e., endovascular vs. surgical - in case an aneurysm was identified), the need for intracranial pressure (ICP) monitoring, brain tissue oxygenation monitoring (PbtO_2_), continuous EEG and external ventricular derivation (EVD) were recorded.

Daily treatments, including the use of vasopressors or inotropic agents, extracorporeal membrane oxygenation (ECMO) and continuous renal replacement therapy (CRRT) were recorded. We also recorded the development of brain-specific complications, such as: rebleeding, intracranial hypertension (ICHT), cerebral vasospasm, DCI, hydrocephalus and seizures. Definition of such complications have been reported elsewhere [27]. We also collected data regarding ICHT management (i.e. osmotic therapy, decompressive craniectomy, hypothermia, barbiturates, hyperventilation). Also, data on prophylaxis and management of vasospasm (i.e., oral nimodipine, intra-arterial nimodipine, induced hypertension and cerebral angioplasty) were collected. Patients in whom nimodipine prophylaxis had to be interrupted before 21 days from SAH onset (i.e. due to severe hemodynamic instability) were considered as not having received the treatment. Both ICU and hospital mortality and neurological outcome at 3 months were collected. Neurologic outcome was assessed using the Glasgow outcome scale at 3 months (GOS: 1 = Severe injury or death without recovery of consciousness, 2 = Severe damage with prolonged state of unresponsiveness and a lack of higher mental functions, 3 = Severe injury with permanent need for help with daily living, 4 = No need for assistance in everyday life, employment is possible but may require special equipment, 5 = Light damage with minor neurological and psychological deficits), which was routinely collected in the follow-up visit or estimated from medical reports. Unfavorable neurological outcome (UO) was defined as GOS 1–3. To allow the analysis of mortality and UO over time, we separated different years of admission into 4 different periods: (1) 2004–2007; (2) 2008–2011; (3) 2012–2015; (4) 2016–2018. We grouped together the years with similar SAH management strategies and practices in our center. Patients’ management has been previously described [27].

### Outcomes

Primary outcomes were hospital mortality rates and neurological outcome assessed by GOS at 3 months of each year from 2004 to 2018 and during the four periods described above.

Secondary outcomes included : (a) the identification of possible predictors of hospital mortality and unfavorable neurological outcome in 3 months; (b) Differences in outcome (mortality and neurological outcome in 3 months) of patients with WFNS 5 compared to WFNS 4; (c) impact of hydrocephalus and intraparenchymal hematoma on patients’ outcome.

### Statistical analysis

Descriptive statistics were computed for all study variables. Categorical data are presented as both as numbers and as percentages. Continuous data are presented as mean (± standard deviation) or median (25th − 75th percentiles), according to the distribution pattern of each variable. Differences between groups were assessed using a χ-square or Fisher’s exact test for categorical variables. For normally distributed continuous variables we used t-Student or ANOVA and, for asymmetrically distributed continuous variables, we applied the Mann-Whitney test or Kruskal-Wallis test. A Cox regression model was used to calculate the hazard ratios (HR) and 95 % confidence intervals (CIs) for factors related to hospital mortality. Co-linearity was checked before modelling. The following variables were included in the model: period of time (2004–2007; 2008–2011; 2012 − 2005; 2016–2018), WFNS score, hydrocephalus and intraparenchymal hematoma and adjusted for known factors associated with mortality. Multivariable logistic regression was performed to calculate adjusted odds ratios (ORs) with 95 % CIs of poor neurological outcome. Co-linearity between variables was checked before modeling. The following variables were included in the model: period of time (2004–2007; 2008–2011; 2012 − 2005; 2016–2018), WFNS score, hydrocephalus and intraparenchymal hematoma and were adjusted by known factors related to poor neurological outcome. We used the Hosmer-Lemeshow test to assess the goodness-of-fit of the model. A p < 0.05 was considered as statistically significant. Statistical analyses were performed using IBM SPSS Statistics 26.

## Results

### Study population

**Fig. 1:**
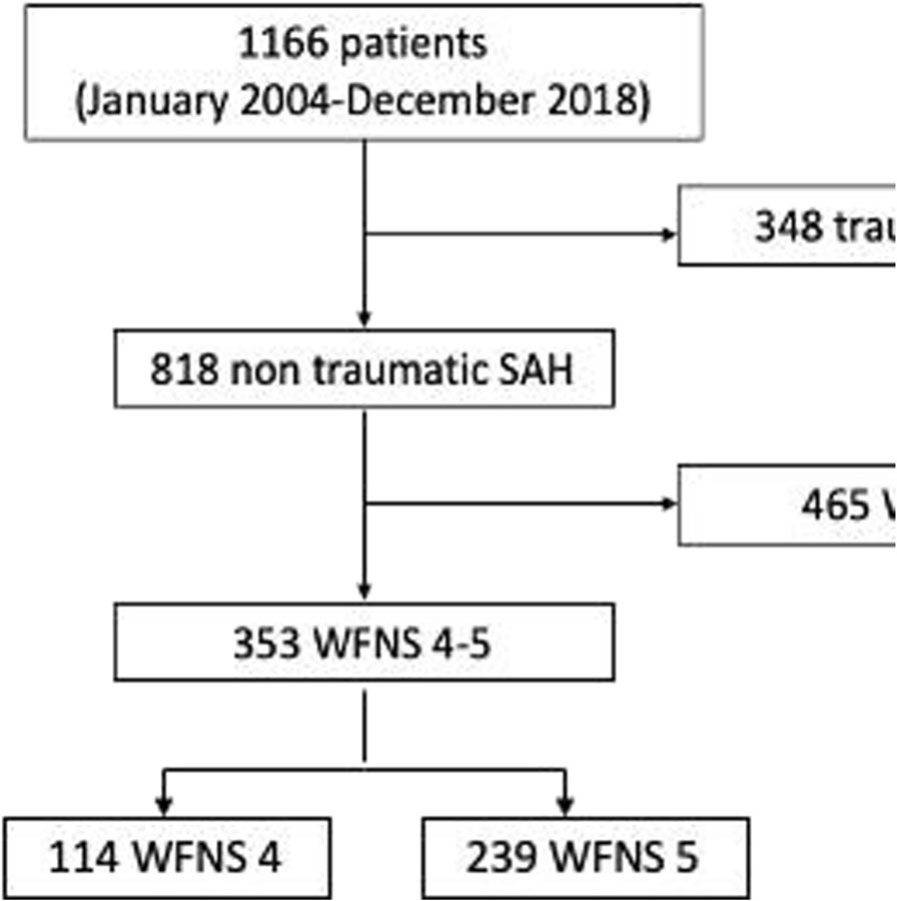
Flow-chart of the study. SAH: subarachnoid hemorrhage; WFNS: World Federation of Neurological Surgeons

On a total of 1166 SAH admitted to the ICU over the study period, 348 were of traumatic origin and 465 had a WFNS of 1 to 3 on admission, leaving 353 (30 %) patients eligible for the final analysis (Fig. 1). Of those 114 (32 %) patients were classified as WFNS 4 and 239 (68 %) patients as WFNS 5. An aneurysm was identified in 298 (84 %) patients; 8 (2 %) patients had an arterio-venous malformation and no cause was identified in 48 patients (14 %). Of those 114 (32 %) patients were classified as WFNS 4 and 239 (68 %) patients as WFNS 5. Overall hospital mortality was 57 % (202/353); of those, 39 (19 % of all deaths) were WFNS 4 and 163 (81 % of all deaths) were WFNS 5. UO was observed in 260 patients (74 %); of those, 61 (24 % of all UO) were WFNS 4 and 199 (77 % of all UO) were WFNS 5.

### Characteristics of the study population over time

Characteristics of the study population are shown in Table 1. Severity of disease, as indicated by the APACHE II and SOFA scores on admission, significantly increased over time. Also, the overall Glasgow Coma Scale was lower in the third and fourth period compared to others. There was a progressive decrease over time in the number of patients having previous neurological disease, cancer, smoking or drug abuse; however, the number of patients being treated with vasopressors increased over time as well as the proportion of patients with hydrocephalus on admission, in particular in the third period. The use of CRRT was rare (< 2 % over time). The proportion of patients with WFNS 5 increased significantly over time. The use of multimodal neuromonitoring and the intensity of therapies also increased over time.

**Table 1:** Characteristic of the population with poor grade subarachnoid hemorrhage (SAH) during the four different study periods. Data are presented as mean ($\pm$SD), median (IQRs) or counts (%)

	All patients N = 353	2004–2007 (N = 69)	2008–2011 (N = 71)	2012–2015 (N = 116)	2016–2018 (N = 97)	p value
Age, years	57 (±14)	60 (± 16)	56 (± 11)	57 (± 14)	54 (± 15)	0.08
Male gender, n (%)	149 (42)	29 (42)	26 (37)	48 (42)	46 (47)	0.57
APACHE score	18 (16–21)	16 (13–19)	18 (15–20)	19 (17–21)	21 (18–24)	< 0.001
SOFA score	8 (5–10)	6 (4–7)	6 (5–9)	8 (6–10)	9 (7–10)	< 0.001
Glasgow coma scale	4 (3–7)	6 (3–10)	7 (3–9)	3 (3–6)	3 (3–6)	< 0.001
WFNS 5, n (%)	239 (68)	34 (49)	38 (54)	92 (79)	75 (77)	< 0.001
Fisher scale 3 or 4 points, n (%)	330 (94)	64 (93)	70 (99)	115 (99)	81 (84)	0.03
Aneurysm, n (%)	288 (82)	54 (78)	56 (79)	93 (80)	85 (88)	0.34
Hypertension, n (%)	146 (41)	35 (51)	19 (27)	51(44)	41(42)	0.03
Diabetes mellitus, n (%)	35 (10)	9 (13)	9 (13)	9 (8)	8 (8)	0.52
Heart disease, n (%)	52 (15)	13 (19)	10 (14)	17 (15)	12 (12)	0.71
Previous neurological disease, n (%)	44 (12)	13 (19)	4 (6)	20 (17)	7 (7)	0.02
Chronic kidney disease, n (%)	7 (2)	1 (1)	0	2 (2)	4 (4)	0.28
COPD, n (%)	27 (8)	4 (6)	5 (7)	8 (7)	10 (10)	0.70
Corticosteroid use, n (%)	13 (4)	1 (1)	3 (4)	4 (3)	5 (5)	0.65
Cancer, n (%)	30 (9)	12 (17)	4 (6)	8 (7)	6 (6)	0.03
Cirrhosis, n (%)	8 (2)	2 (3)	1 (1)	2 (2)	3 (3)	0.85
Alcohol, n (%)	64 (18)	22 (32)	11 (16)	13 (11)	18 (19)	0.005
Smoking, n (%)	79 (22)	35 (51)	5 (7)	20 (17)	19 (20)	< 0.001
Drug abuse, n (%)	22 (6)	15 (22)	1 (1)	3 (3)	3 (3)	< 0.001
Support Therapies						
Vasopressor use, n (%)	256 (73)	41 (60)	52 (73)	77 (66)	86 (89)	0.001
Inotropic use, n (%)	55 (16)	1 (1)	9 (13)	14 (12)	31 (32)	< 0.001
ECMO v-v, n (%)	3 (1)	0	0	1 (1)	2 (2)	0.41
Mechanical ventilation, n (%)	321 (91)	59 (86)	65 (92)	108 (93)	89 (92)	0.36
CRRT, n (%)	3 (1)	1 (1)	0	1 (1)	1 (1)	0.82
Neurological monitoring and specific treatments						
Surgical treatment (clipping), n (%)	55 (16)	11 (16)	11 (16)	14 (12)	19 (20)	0.52
Endovascular treatment, n (%)	162 (46)	31 (45)	40 (56)	45 (39)	46 (47)	0.13
ICP monitoring, n (%)	264 (75)	49 (71)	56 (79)	80 (69)	47 (49)	0.14
PbtO_2_ monitoring, n (%)	49 (14)	0	0	2 (2)	47 (49)	< 0.001
Continuous EEG monitoring, n (%)	220 (63)	42 (61)	52 (73)	62 (53)	64 (66)	0.04
Vasospasm prophylaxis, n (%)	235 (67)	53 (77)	58 (82)	75 (65)	49 (51)	< 0.001
Hyperventilation, n (%)	168 (48)	29 (42)	35 (49)	57 (49)	47 (49)	0.78
Osmotic therapy, n (%)	158 (45)	30 (44)	22 (31)	58 (50)	48 (50)	0.05
Decompressive craniectomy, n (%)	14 (4)	2 (3)	3 (4)	3 (3)	6 (6)	0.56
Barbituric coma, n (%)	68 (19)	13 (19)	13 (18)	21 (18)	21 (22)	0.92
Hypothermia, n (%)	46 (13)	2 (3)	4 (6)	14 (12)	26 (27)	< 0.001
Induced hypertension, n (%)	128 (36)	35 (51)	21 (30)	33 (28)	39 (40)	0.01
Intra-arterial injection of nimodipine, n (%)	37 (11)	1 (1)	7 (10)	6 (5)	23 (24)	< 0.001
Neurological complications						
Intraparenchymal hematoma, n (%)	153 (43)	39 (57)	25 (35)	41 (35)	48 (50)	0.01
Seizure, n (%)	94 (27)	14 (20)	26 (37)	26 (22)	28 (29)	0.10
Rebleeding, n (%)	27 (8)	7 (11)	1 (1)	7 (6)	12 (12)	0.05
Hydrocephalus, n (%)	150 (43)	22 (32)	14 (20)	75 (65)	39 (40)	0.001
Delayed cerebral ischemia, n (%)	82 (23)	27 (39)	15 (21)	17 (15)	23 (24)	0.002
Intracranial hypertension, n (%)	230 (65)	48 (70)	42 (59)	80 (69)	60 (62)	0.40
Outcomes						
UO, n (%)	260 (74)	51 (74)	50 (70)	85 (73)	90 (77)	0.86
GOS at 3 months, median (IQR)	1 (1–4)	1 (1–4)	2 (1–4)	1 (1–4)	1 (1–3)	0.42
ICU LOS- days, median (IQR)	8 (2–7)	7 (2–15)	10 (3–16)	6 (1–16)	9 (2–21)	0.11
Hospital LOS- days, median (IQR)	13 (2–35)	9 (2–34)	13 (3–35)	10 (1–28)	20 (2–44)	0.06
ICU mortality, n (%)	195 (56)	43 (62)	33 (47)	70 (60)	49 (51)	0.13
Hospital mortality, n (%)	202 (57)	45 (65)	35 (49)	73 (63)	49 (51)	0.07

*APACHE score* Acute Physiology and Chronic Health Evaluation; *SOFA score* Sequential Organ Failure Assessment; *GCS* Glasgow coma scale; *WFNS* World Federation of Neurological surgeons; *ICU* Intensive Care Unit; *SD* standard deviation; *IQR* interquartile range; *ECMO v-v* venous-venous extracorporeal membrane oxygenation; *CRRT* renal replacement therapy; *ICP* intracranial pressure; *PbtO*_*2*_ partial pressure of brain tissue oxygen; *EEG* electroencephalogram; *LOS* length of stay

### Changes in mortality and neurological outcome over time

**Fig. 2:**
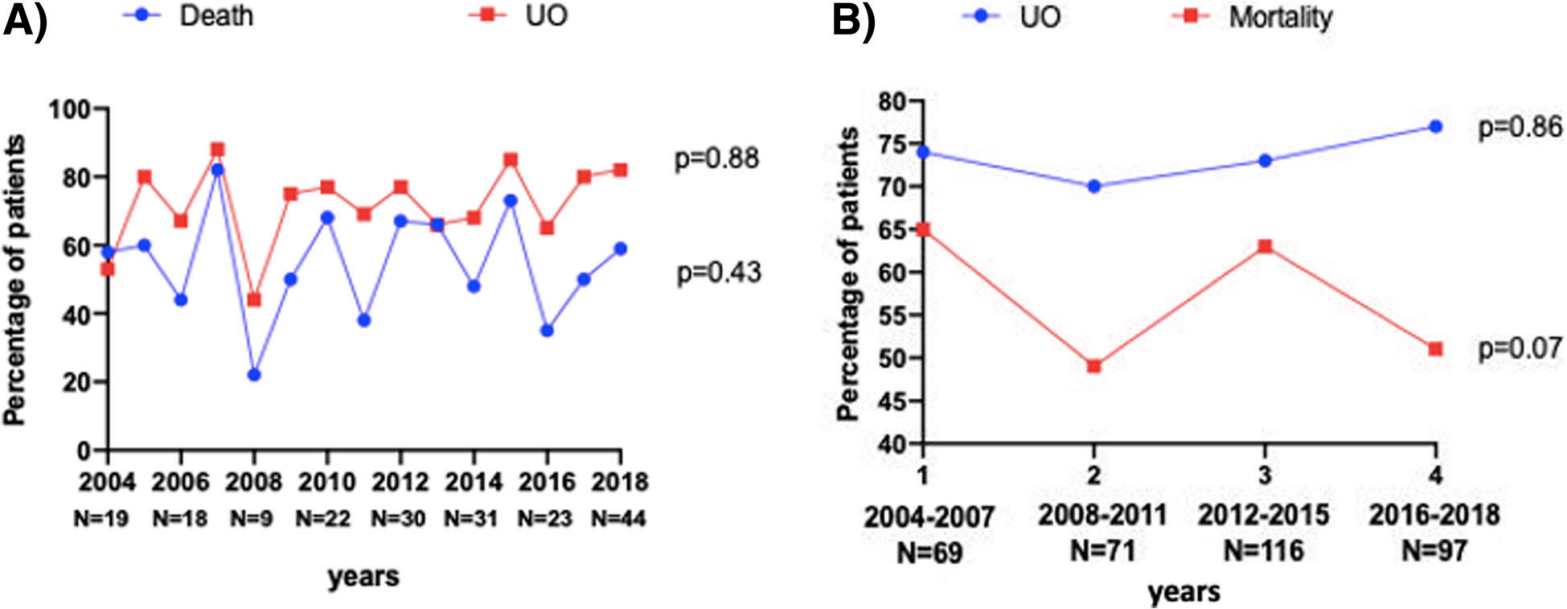
Panel **a**) Proportion of non-survivors and of patients with UO over the years from 2004-2018. Panel **b**) Proportion of non-survivors and of patients with UO into the 4 study periods. *P*-values were calculated using qui-square test comparing the proportion of non survivors/ patients with poor outcome over time

Hospital mortality and the percentage of patients with unfavorable neurological outcome did not vary significantly over the years as shown in Fig. 2b. When analyzing survival data according to year groups there was a reduction in hospital mortality in the second and fourth period but not in the third period, where the mortality rate is similar to baseline (2004–2007: 45/69, 65 %; 2008–2011: 35/71, 49 %; 2012–2015: 73/116; 63 %; 2016–2018: 49/97, 51 %; p = 0.07 – Fig. 2b). Non survivors were older, had a worse neurological status at presentation and were more severely ill, as demonstrated by the APACHE and SOFA score on admission. These patients also had more comorbidities and developed more neurological complications such as rebleeding, hydrocephalus and intracranial hypertension (Supplemental Table S1). In the Cox regression model, being admitted in 2008–2011 (HR 0.55 [95 % CI 0.34–0.89]) and 2016–2018 (HR 0.33 [95 % CI 0.20–0.53]) compared to being admitted in 2004–2007 were associated with a lower risk of mortality (Table 2).

**Table 2:** Cox regression for factors associated with hospital mortality

	Univariable analysis HR (95 % CI)	P value	Multivariable analysis HR (95 % CI)	P value
Age	1.02 (1.01–1.03)	0.002	**1.02 (1.00-1.03)**	**0.022**
SOFA score	1.18 (1.12–1.24)	0.001	**1.10 (1.03–1.17)**	**0.006**
WFNS 5	2.80 (1.97–3.98)	0.001	**2.12 (1.43–3.14)**	**0.001**
Endovascular treatment	0.34 (0.25–0.45)	0.001	**0.51 (0.36–0.73)**	**0.001**
Prophylactic nimodipine	0.29 (0.22–0.38)	0.001	**0.50 (0.35–0.72)**	**0.001**
Intracranial hypertension	4.80 (3.23–7.12)	0.001	**3.56 (2.33–5.41)**	**0.001**
Rebleeding	2.09 (1.35–3.23)	0.001	1.39 (0.86–2.24)	0.174
Hydrocephalus	0.63 (0.47–0.84)	0.001	**0.60 (0.43–0.84)**	**0.002**
Seizures	0.60 (0.43–0.84)	0.003	0.84 (0.58–1.20)	0.316
Diabetes mellitus	0.56 (0.32–0.96)	0.035	0.72 (0.41–1.26)	0.254
Heart disease	1.45 (1.01–2.07)	0.045	1.36 (0.90–2.06)	0.151
Intraparenchymal hematoma	1.01 (0.76–1.33)	0.951	0.91 (0.67–1.23)	0.545
2008–2011	0.68 (0.44–1.06)	0.090	**0.55 (0.34–0.89)**	**0.015**
2012–2015	1.01 (0.69–1.46)	0.981	0.71 (0.46–1.09)	0.117
2016–2018	0.69 (0.46–1.03)	0.072	**0.33 (0.20–0.53)**	**0.001**

Goodness of fit: p = 0.37; *WFNS* world federation of neurological surgeons; *HR* hazard ratio; *CI* confidence interval. Values in bold represent statistical significance in the multivariable analysis

The proportion of patients with UO remained high over the study period (2004–2007: 51/69, 74 %; 2008–2011: 50/71, 70 %; 2012–2015: 85/116, 73 %; 2016–2018 :90/97, 77 %; p = 0.86 – Fig. 2). In the univariable analysis, the risks of UO, with 2004–2007 as references, did not decrease over time (2008–2011: OR 0.84 [95 % CI 0.40–1.76] − 2012–2015: 0.97 [95 % CI 0.49–1.90] − 2016–2018: OR 1.14 [0.56–2.32]).

Patients with UO at 3 months were older and more severely ill on admission and had a lower initial GCS score when compared to those with FO. They also required more support therapy and experienced more neurological complications such as rebleeding and intracranial hypertension (Supplemental Table S1). In the multivariable analysis (Table 3), the studied period was not associated with UO.

**Table 3:** Logistic regression for associated with poor neurologic outcome in 3 months (GOS 1–3)

	Univariable analysis OR (CI 95 %)	p-value	Multivariable analysis OR (CI 95 %)	p-value
Age	1.02 (1.01–1.04)	0.013	**1.04 (1.01–1.06)**	**0.005**
WFNS 5	4.32(2.62–7.13)	0.001	**3.23 (1.67–6.25)**	**0.001**
Intracranial hypertension	10.14(5.87–17.51)	0.001	**10.15 (5.24–19.66)**	**0.001**
Rebleeding	10.22(1.37–76.43)	0.024	3.77 (0.46–30.56)	0.215
Hydrocephalus	0.64(0.40–1.03)	0.068	0.66 (0.32–1.33)	0.243
Intraparenchymal hematoma	1.02 (0.63–1.64)	0.940	0.90 (0.49–1.67)	0.747
Endovascular treatment	0.31(0.19–0.51)	0.001	0.56 (0.29–1.09)	0.087
Prophylactic nimodipine	9.27(2.20-39.04)	0.001	**0.29 (0.11–0.77)**	**0.013**
2008–2011	0.84 (0.40–1.76)	0.645	1.12 (0.44–2.85)	0.809
2012–2015	0.97 (0.49–1.90)	0.924	0.71 (0.28–1.85)	0.487
2016–2018	1.14 (0.56–2.32)	0.727	1.26 (0.48–3.27)	0.638

Goodness of fit: p = 0.73; *WFNS* world federation of neurological surgeons; values in bold represent statistical significance in the multivariable analysis. Age was included in the model as a continuous variable

### Differences between WFNS 4 and WFNS 5 patients

Patients with WFNS 5 had a higher proportion of severe bleeding (i.e. Fisher scale 3 or 4), developed more frequently intracranial hypertension and required more aggressive therapeutic management than WFNS 4 patients (Supplemental Table S2). Hospital mortality (163/239, 68 % vs. 39/114, 34 %; p < 0.001) and the occurrence of UO (199/239, 83 % vs. 61/114, 54 %; p < 0.001) was higher in WFNS 5 patients than others. In the multivariable analysis WFNS 5 was also associated with an increased risk of hospital death (HR 2.12 [95 % 1.43–3.14]) and of UO (OR 3.23 [1.67–6.25]).

### The impact of hydrocephalus and intraparenchymal hematoma on outcome

Of the 353 patients, 150 (43 %) developed hydrocephalus and were treated with external ventricular drainage. Patients with hydrocephalus had a lower likelihood of mortality (HR 0.60 [95 % CI 0.43–0.84) than others. Hydrocephalus was not associated with neurological outcome (OR 0.66 [95 % CI 0.32–1.33]).

Intraparenchymal hematoma on admission was present in 153/353 (43 %) patients. More than half of these patients (89/153, 58 %) died and 74 % (113/153) had UO; however, intraparenchymal hematoma was not independently associated with neither mortality (HR 0.91 [0.67–1.23]) or UO (OR 0.90 [0.49–1.67]).

**Fig. 3:**
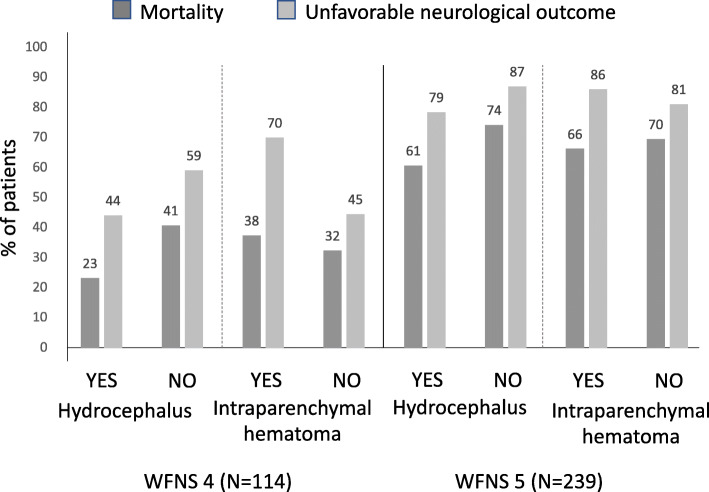
Percentage of mortality and poor neurological outcome according to WFNS score

The combination of WFNS score with hydrocephalus or intraparenchymal hematoma did not influence the higher mortality and UO rates in those with WFNS 5 on admission (Fig. 3).

## Discussion

In this study we have showed that there was no clear trend in reduction of mortality rates over the years and that the occurrence of UO remained high and unchanged in poor grade SAH patients. WFNS 5 and the absence of hydrocephalus were independent predictors of hospital mortality. WFNS 5 was also independently associated with UO. The presence of intracerebral hematoma and hydrocephalus did not significantly influence the high mortality and UO rates in WFNS 5 patients.

Several studies have reported worldwide a decrease in SAH mortality over time, regardless of the outcome used (i.e., at hospital discharge or at 30 days). Indeed, two meta-analysis including studies from Europe, Japan, China, USA, Chile, Australia and New Zealand have reported a decrease in mortality of approximately 0.8 and 0.9 % per year and from 1972 to 2002 and from 1980 to 2005, respectively [28, 29]. In Australia, two retrospective studies have shown a decrease in mortality varying from 0.7 to 2.7 % a year after SAH over time [30, 31]. Similarly, mortality of SAH patients has decreased steadily between 1998 and 2007, regardless of the initial treatment of choice (i.e. surgical clipping or endovascular coiling) [32]. In our study, we found a significant reduction in mortality from 2004 to 2007 to 2008–2011; however, mortality increased in 2012–2015 with similar rates to 2004–2007. The increase in mortality in the third period could be explained by the significant increase in the proportions of WFNS 5 compared to the second period and due to the high proportions of patients with intracranial hypertension. In fact, similar incidences of intracranial hypertension and subsequently mortality rates were observed in the first and third period. In the last studied period (2016–2018) mortality starts to fall again, despite the elevated proportions of WFNS 5 patients. This could be due to the implementation of new neuromonitoring technologies, such as PbtO_2_ catheters, or more aggressive therapies (i.e. intra-arterial vasodilators), which may have contributed to early diagnosis of neurological complications and a more adequate management of refractory cerebral vasospasm. This difference from what was reported in previous studies could be explained because these studies have frequently compared 80’s and 90’s with more recent decades, while we focused our data analysis in 2004–2018, where endovascular and surgical treatment were already well established; also, while previous studies often focused on SAH of all clinical severity on admission [32], we evaluated only poor grade patients, in whom the mortality rate is the highest and relevant improvement in patients’ management might significantly influence patients’ outcome over time. Of course, not all confounders could be adequately assessed in this retrospective longitudinal analysis; moreover, other series of SAH patients have reported unchanged mortality rates over the same period of time [33, 34].

Regarding functional neurological status, a study conducted in the UK including all WFNS grades showed a 50 % decrease in the occurrence of UO, which was assessed by the modified Rankin scale, comparing two distinct time periods (i.e. 1981–1986 vs. 2002–2008) [28]. In the USA, a decrease in the proportion of patients with high disability was observed from 1998 to 2013 [35]. Similarly, in developing countries such as India, there was a significant decrease of patients with UO at 3 months, which was assessed by GOS, from 1996–2015; one of the main explanations for such findings was the improvement of neurosurgical services and overall therapies in this country [36]. However, when only patients with poor grade clinical status were considered, early interventions and aggressive treatment did not significantly reduce the high number of patients who were discharged with severe disability from the hospital [7, 37, 38]. A meta-analysis also showed that in poor grade SAH patients there was an initial improvement of outcome form 70’s to 90’ but that there has been no further gain in terms of neurological recovery thereafter; this could be explained by the high proportion of WFNS 5 patients included in the studies [20]. Our study reported similar results and underlined a very high occurrence of UO in WFNS 5 patients.

Our findings suggest that patients with poor grade SAH may not be an homogenous group; in particular, patients with WFNS 5 have a worse outcome than those with WFNS 4. This has been previously suggested in other studies [7, 20, 39], but our study was designed to specifically investigate the differences in outcome between WFNS 4 and 5. The highest mortality and UO rates observed in WFNS 5 patients might be due to several factors, including the extension of the initial injury, the severity of bleeding as well as the occurrence of early (i.e., intracranial hypertension) and late (i.e., DCI) brain complications. Future trials should focus on the pathophysiological mechanisms as the response to therapies of WFNS 4 and WFNS 5 patients separately to optimize therapeutic interventions in such patients, better stratify for the severity of disease and more accurately prognosticate their outcome.

We also investigated additional factors that could further aggravate or influence the poor outcome of WFNS 5 patients, such as hydrocephalus and the presence of intraparenchymal hematoma. We found that acute hydrocephalus did not increase the chance of death, possibly because clinical deterioration associated with hydrocephalus could promptly be treated with external ventricular derivation, which in many cases may resolve symptoms [40]. In our cohort, all patients who had hydrocephalus were treated with external ventricular derivation (EVD); however, we do not have data regarding any neurological improvement after EVD insertion and we can only speculate that this would be the reason explaining why hydrocephalus was associated with a better neurological recovery. As such, the importance of early recognition and treatment of this condition should be highlighted and reported in future descriptive and interventional studies. As another study reported that hydrocephalus after SAH was associated with UO [41], this complication should be further studied in multicentric cohorts. In our study, we also reported a high prevalence of intraparenchymal hematoma. However, this had no impact on mortality or UO. A possible explanation is that hematoma evacuation surgery was performed in 82 % of patients and this might have reduced the risk of secondary brain injury [22, 42]. Also, it is possible that the association of intraparenchymal hematoma with UO reported in other studies might be related to the decision of limiting aggressive therapies, including surgical drainage. Moreover, previous studies included all WFNS categories, while we specifically focused only on “poor grade” patients [22, 43–45]. Poor grade SAH is associated with intraparenchymal hematoma [42, 43, 45]. Also, a study by Wan et al. have shown that while intraparenchymal hematoma is an important predictor of mortality after SAH, the significance of this association was reduced when adjusted by the initial WFNS score [43].

Potential limitations of the present study should be taken consideration. First, many additional variables could not be collected as data availability and quality can be challenging in retrospective studies covering a long period of time. Also, decision of specific therapies could have been influenced by the patient status, physician or family decisions and not solely on patient’s severity which may have affected outcome. Finally, the follow-up period for neurological outcome could have been prolonged to 6–12 months to better investigate the long-term evolution of such poor grade SAH patients.

## Conclusions

In this study, we have demonstrated that both mortality and unfavorable neurological outcome remained frequent in poor grade SAH patients. We have also shown that poor grade patients are not a homogenous group, and that patients with an initial WFNS grade of 5 experience worse prognosis than other poor grade patients; this should be taken into consideration for future clinical studies. Post-SAH acute hydrocephalus, when treated early, may be associated with reduced mortality in poor grade patients.

## Supplementary Information

Additional file 1

Additional file 2

## Data Availability

The datasets used and/or analyzed during the current study available from the corresponding author on reasonable request.

## Abbreviations

APACHE: Acute physiology and chronic health evaluation
CI: Confidence interval
COPD: Chronic obstructive pulmonary disease
CRRT: continuous renal replacement therapy
CT: Computed tomography
DCI: Delayed cerebral ischemia
ECMO: Extracorporeal membrane oxygenation.
EVD: External ventricular derivation
GCS: Glasgow coma scale
GOS: Glasgow outcome scale
HR: Hazard ration
ICHT: Intracranial hypertension
ICP: Intracranial pressure
ICU: Intensive Care Unit
OR: Odds ratio
SAH: Subarachnoid hemorrhage
SOFA: Sequential organ failure assessment
UO: Unfavorable outcome
WFNS: World Federation of Neurological Surgeons
