# Supplementary material for: Time course of outcome in poor grade subarachnoid hemorrhage patients: a longitudinal retrospective study
Source: BMC Neurol. 2021 May 13;21:196. doi: 10.1186/s12883-021-02229-1 (PMC8117582; doi:10.1186/s12883-021-02229-1)
Supplement: Supplementary file 1 — Additional file 1 [file 12883_2021_2229_MOESM1_ESM.docx]

**Supplemental Table S1**: Characteristics of patients according to hospital mortality and neurologic outcome at 3 months (FO = favorable; UO = unfavorable). Data are presented as mean ($\pm$SD), median (IQRs) or counts (%).

|  | Survivors  N=151 | Non-survivors  N= 202 | p value | FO  N=93 | UO  N= 206 | p value |
| --- | --- | --- | --- | --- | --- | --- |
| Age, years | 55 (±13) | 58 (±15) | 0.03 | 53 (±13) | 58 (±14) | 0.01 |
| Male gender, n (%) | 65 (43) | 84 (42) | 0.83 | 43 (46) | 106 (41) | 0.39 |
| APACHE score | 17(13-19) | 20 (17-23) | 0.001 | 16 (12-19) | 19 (17-22) | 0.001 |
| SOFA score | 6 (4-9) | 9 (6-10) | 0.001 | 6 (3-8) | 8 (6-10) | 0.001 |
| GCS on admission | 7 (3-9) | 3 (3-5) | 0.001 | 7 (5-10) | 3 (3-6) | 0.001 |
| Fisher scale 3 or 4, n (%) | 139 (92) | 191 (95) | 0.39 | 84 (90) | 246 (95) | 0.39 |
| WFNS 5, n (%) | 76 (50) | 163 (81) | 0.001 | 40 (43) | 199 (77) | 0.001 |
| Aneurysm, n (%) | 124 (82) | 164 (81) | 0.89 | 77 (83) | 211 (81) | 0.88 |
| Hypertension, n (%) | 77 (51) | 69 (34) | 0.002 | 40 (43) | 160 (62) | 0.71 |
| Diabetes mellitus, n (%) | 21 (14) | 14 (7) | 0.05 | 9 (10) | 26 (10) | 0.99 |
| Heart disease, n (%) | 16 (11) | 36 (18) | 0.07 | 9 (10) | 43 (17) | 0.13 |
| Previous neurological disease, n (%) | 20 (13) | 24(12) | 0.75 | 13 (14) | 31 (12) | 0.60 |
| CKD, n (%) | 4 (3) | 3 (2) | 0.47 | 3 (3) | 4 (2) | 0.99 |
| COPD, n (%) | 7 (5) | 20 (10) | 0.70 | 7 (8) | 20 (8) | 0.99 |
| Corticosteroid use, n (%) | 6 (4) | 7 (4) | 0.79 | 3 (3) | 10 (4) | 0.99 |
| Cancer, n (%) | 11 (7) | 19 (9) | 0.57 | 7 (8) | 23 (9) | 0.83 |
| Cirrhosis, n (%) | 1 (1) | 7 (3) | 0.15 | 0 | 8 (3) | 0.12 |
| Alcohol, n (%) | 32 (21) | 32 (16) | 0.21 | 24 (26) | 40 (15) | 0.03 |
| Smoking, n (%) | 39 (26) | 40 (20) | 0.20 | 29 (32) | 50 (19) | 0.02 |
| Drug abuse, n (%) | 9 (6) | 13 (6) | 0.99 | 7 (8) | 15 (7) | 0.62 |
| Surgical treatment, n (%) | 22 (15) | 33 (16) | 0.77 | 12 (13) | 43 (21) | 0.51 |
| Endovascular treatment, n (%) | 98 (65) | 64 (32) | 0.001 | 62 (67) | 100 (39) | 0.001 |
| Intraparenchymal hematoma, n (%) | 64 (42) | 89 (44) | 0.83 | 40 (43) | 113 (44) | 0.99 |
| Seizure, n (%) | 50 (33) | 44 (22) | 0.02 | 27 (29) | 67 (26) | 0.59 |
| Rebleeding | 4 (3) | 23 (11) | 0.002 | 1 (1) | 26 (10) | 0.003 |
| Hydrocephalus, n (%) | 75 (50) | 75 (37) | 0.02 | 47 (51) | 103 (40) | 0.09 |
| Delayed cerebral ischemia, n (%) | 39 (26) | 43 (21) | 0.37 | 17 (18) | 65 (25) | 0.20 |
| Intracranial hypertension, n (%) | 57(38) | 173 (86) | 0.001 | 25 (27) | 205 (79) | 0.001 |
| Vasopressor use n (%) | 98 (65) | 158 (78) | 0.008 | 51 (55) | 205 (79) | 0.001 |
| Inotropic use, n (%) | 15 (10) | 40 (20) | 0.001 | 4 (4) | 51 (20) | 0.001 |
| ECMO, n (%)^#^ | 0 | 3 (2) | 0.26 | 0 | 3 (1) | 0.57 |
| Mechanical ventilation, n (%) | 125 (83) | 196 (97) | 0.001 | 74 (80) | 247 (95) | 0.001 |
| CRRT, n (%) | 0 | 3 (2) | 0.26 | 0 | 3 (1) | 0.57 |
| ICP monitoring, n (%) | 123 (82) | 141 (70) | 0.01 | 72 (77) | 192 (74) | 0.58 |
| PbtO_2_ monitoring, n (%) | 23 (15) | 26 (13) | 0.54 | 9 (10) | 40 (15) | 0.22 |
| Continuous EEG monitoring, n (%) | 123 (82) | 97 (48) | 0.001 | 67 (72) | 153 (59) | 0.03 |
| Prophylactic nimodipine, n (%) | 123 (82) | 112 (55) | 0.001 | 78 (84) | 157(60) | 0.001 |
| Hyperventilation, n (%) | 37 (25) | 131 (65) | 0.001 | 12 (13) | 156 (60) | 0.001 |
| Osmotic therapy, n (%) | 32 (21) | 126 (62) | 0.001 | 9 (10) | 149 (53) | 0.001 |
| Decompressive craniectomy, n (%) | 5 (3) | 9 (5) | 0.78 | 2 (2) | 12 (5) | 0.37 |
| Barbituric coma, n (%) | 12 (8) | 56 (28) | 0.001 | 3 (3) | 65 (25) | 0.001 |
| Hypothermia, n (%) | 10 (7) | 36 (18) | 0.002 | 2 (2) | 44 (14) | 0.001 |
| Induced hypertension, n (%) | 66 (44) | 62 (31) | 0.01 | 38 (41) | 90 (35) | 0.32 |
| Intra-arterial nimodipine, n (%) | 21 (14) | 16 (8) | 0.08 | 10 (11) | 27 (10) | 0.99 |
| ICU LOS- days, median (IQR) | 16 (9-23) | 2 (1-9) | 0.001 | 15 (7-19) | 5 (1-4) | 0.001 |
| Hospital LOS- days, median (IQR) | 38 (25-79) | 2 (1-9) | 0.001 | 29 (22-56) | 6 (1-23) | 0.001 |

APACHE score: Acute Physiology and Chronic Health Evaluation; SOFA score: Sequential Organ Failure Assessment; GCS: Glasgow coma scale; WFNS: World Federation of Neurological surgeons; ICU: Intensive Care Unit; SD: standard deviation; IQR: interquartile range; ECMO v-v: venous-venous extracorporeal membrane oxygenation; CRRT: renal replacement therapy; ICP: intracranial pressure; PbtO_2_ : partial pressure of brain tissue oxygen; EEG: electroencephalogram; LOS: length of stay.
