# Supplementary material for: Time course of outcome in poor grade subarachnoid hemorrhage patients: a longitudinal retrospective study
Source: BMC Neurol. 2021 May 13;21:196. doi: 10.1186/s12883-021-02229-1 (PMC8117582; doi:10.1186/s12883-021-02229-1)
Supplement: Supplementary file 2 — Additional file 2 [file 12883_2021_2229_MOESM2_ESM.docx]

**Supplemental Table S2.** Characteristics of the study population, according to the initial WFNS score. Data are presented as mean ($\pm$SD), median (IQRs) or counts (%).

|  | WFNS 4  N=114 | WFNS 5  N= 239 | p value |
| --- | --- | --- | --- |
| Age, years | 57 (±15) | 56 (±13) | 0.90 |
| Male gender, n (%) | 45 (40) | 104 (44) | 0.50 |
| APACHE score | 14 (12-17) | 20 (17-23) | 0.77 |
| SOFA score | 5 (3-7) | 9 (6-10) | 0.001 |
| Glasgow coma scale on admission | 9 (7-11) | 3 (3-4) | 0.001 |
| Fisher scale 3 or 4, n (%) | 102 (90) | 228 (95) | 0.04 |
| Aneurysm, n (%) | 92 (81) | 196 (82) | 0.77 |
| Hypertension, n (%) | 56 (49) | 90 (38) | 0.05 |
| Diabetes mellitus, n (%) | 16 (14) | 19 (8) | 0.90 |
| Heart disease, n (%) | 19 (17) | 33 (14) | 0.52 |
| Previous neurological disease, n (%) | 18 (16) | 26 (11) | 0.30 |
| Chronic kidney disease, n (%) | 4 (4) | 3 (1) | 0.30 |
| COPD, n (%) | 5 (4) | 22 (9) | 0.14 |
| Corticosteroid use, n (%) | 4 (4) | 9 (4) | 0.99 |
| Cancer, n (%) | 12 (11) | 18 (8) | 0.41 |
| Cirrhosis, n (%) | 4 (4) | 4 (2) | 0.28 |
| Alcohol, n (%) | 31 (27) | 33 (14) | 0.003 |
| Smoking, n (%) | 33 (29) | 46 (19) | 0.06 |
| Drug abuse, n (%) | 12 (11) | 10 (4) | 0.30 |
| Surgical treatment (clipping), n (%) | 22 (19) | 33(14) | 0.21 |
| Endovascular treatment, n (%) | 60 (53) | 102 (43) | 0.99 |
| Intraparenchymal hematoma, n (%) | 46 (40) | 107 (45) | 0.49 |
| Seizure, n (%) ^*^ | 44 (39) | 50 (21) | 0.001 |
| Re-bleeding, n (%)^*^ | 5 (4) | 22 (9) | 0.14 |
| Hydrocephalus, n (%) ^*^ | 43 (38) | 107 (45) | 0.30 |
| Delayed cerebral ischemia, n (%) ^*^ | 33 (29) | 49 (21) | 0.08 |
| Intracranial hypertension, n (%) ^*^ | 51 (45) | 179 (75) | 0.001 |
| Vasopressor use, n (%) ^#^ | 68 (60) | 188 (79) | 0.001 |
| Inotropic use, n (%) ^#^ | 8 (7) | 47 (20) | 0.002 |
| ECMO, n (%)^#^ | 0 | 3 (1) | 0.55 |
| Mechanical ventilation, n (%) ^#^ | 88 (77) | 233 (98) | 0.001 |
| CRRT, n (%) ^#^ | 1 (1) | 2 (1) | 0.99 |
| ICP monitoring, n (%) | 85 (75) | 179 (75) | 0.99 |
| PbtO_2_ monitoring, n (%) | 8 (7) | 41 (17) | 0.01 |
| Continuous EEG monitoring, n (%) | 86 (75) | 134 (57) | 0.001 |
| Vasospasm prophylaxis, n (%) | 90 (79) | 145 (61) | 0.001 |
| Hyperventilation, n (%) | 35 (31) | 133 (56) | 0.001 |
| Osmotic therapy, n (%) | 30 (26) | 128 (54) | 0.001 |
| Decompressive craniectomy, n (%) | 2 (2) | 12 (5) | 0.24 |
| Barbituric coma, n (%) | 15 (13) | 53 (22) | 0.06 |
| Hypothermia, n (%) | 35 (31) | 133 (56) | 0.001 |
| Induced hypertension, n (%) | 51 (45) | 77 (32) | 0.03 |
| ICU LOS, days | 11 (5-17) | 5 (1-16) | 0.001 |
| Hospital LOS, days | 23 (11-43) | 6 (1-29) | 0.001 |
| GOS at 3 months | 3 (1-5) | 1 (1-3) | 0.001 |
| UO, n (%) | 61 (54) | 199 (83) | 0.001 |
| ICU mortality, n (%) | 35 (31) | 160 (67) | 0.001 |
| Hospital mortality, n (%) | 39 (34) | 163 (68) | 0.001 |

APACHE score: Acute Physiology and Chronic Health Evaluation; SOFA score: Sequential Organ Failure Assessment; GCS: Glasgow coma scale; WFNS : World federation of neurological surgeons; ICU: Intensive Care Unit; SD: standard deviation; IQR: interquartile range; ECMO v-v: venous-venous extracorporeal membrane oxygenation ; CRRT : continuous renal replacement therapy; ICP: intracranial pressure; PbtO_2_ : partial pressure of brain tissue oxygen; EEG: electroencephalogram; LOS: length of stay; UO: unfavorable outcome; GOS: Glasgow outcome scale.
